# Qualitative nursing research: evidence of scientific validation from a translational perspective

**DOI:** 10.17533/udea.iee.v42n1e11

**Published:** 2024-04-29

**Authors:** João Cruz, Ainoã de Oliveira Lima, Edmara Chaves Costa

**Affiliations:** 2 Registered Nurse, Master’s Student in Nursing. Email: ainoaoliveiralima@outlook.com University for International Integration of the Afro-Brazilian Lusophony Brazil ainoaoliveiralima@outlook.com; 3 Veterinarian, Ph.D., Assistant Professor. Email: edmaracosta@unilab.edu.br University for International Integration of the Afro-Brazilian Lusophony Brazil; 4 Institute of Health Sciences, University for International Integration of the Afro-Brazilian Lusophony, Redenção (Brazil) University for International Integration of the Afro-Brazilian Lusophony Brazil

**Keywords:** qualitative research, validation study, nursing theory, nursing., investigación cualitativa, estudio de validación, teoría de enfermería, enfermería., pesquisa qualitativa, estudo de validação, teoria de enfermagem, enfermagem.

## Abstract

This article aims to reflect on scientific validation strategies in qualitative research in the light of translational theory in nursing. It is a reflection based on translational theory applied to nursing in strategies for validating qualitative studies. From this angle, validation is recognized as an adaptable construct, capable of eliciting/favoring an understanding of the subjectivity of the target audience in its relationship with the object of interest/study/research. The potential for advancing the science-profession lies in the interdisciplinary confluence of validation mechanisms, qualitative studies, the translational perspective, and nursing research. This confluence has the capacity to extend beyond theoretical and epistemological aspects. However, it is crucial to emphasize its profound, expressive, and relevant impact on the construction of scientific evidence. This impact aims to enhance the rigor and reliability of qualitative research, thereby bolstering its credibility and applicability in clinical practice.

## Resumen

El objetivo de este artículo es reflexionar sobre las estrategias de validación científica en la investigación cualitativa a la luz de la teoría traslacional en enfermería. Se trata de una reflexión basada en la teoría traslacional aplicada a la enfermería y en las estrategias de validación de los estudios cualitativos. Desde este ángulo, la validación se reconoce como un constructo adaptable, capaz de favorecer la comprensión de la subjetividad del público objetivo en su relación con el objeto de investigación. Esta confluencia interdisciplinar entre los mecanismos de validación, los estudios cualitativos, la perspectiva traslacional y la investigación en enfermería tiene el potencial de contribuir al avance de la ciencia-profesión con un alcance más allá de los aspectos teóricos y epistemológicos, pero desde la perspectiva de un impacto profundo, expresivo y relevante en la construcción de la evidencia científica con aún mayor rigor y fiabilidad en la investigación cualitativa y, por tanto, a su credibilidad y aplicabilidad en la práctica clínica.

## Resumo

O artigo tem por objetivo refletir sobre as estratégias de validação científica em pesquisas de abordagem qualitativa à luz da teoria translacional em enfermagem. Trata-se de uma reflexão que tem como fundamento a teoria translacional aplicada à enfermagem em estratégias de validação de estudos qualitativos. Por esse ângulo, a validação é reconhecida como um constructo adaptável, capaz de suscitar/favorecer a compreensão da subjetividade do público-alvo na sua relação com o objeto de interesse/estudo/pesquisa. Esta confluência interdisciplinar entre os mecanismos de validação, os estudos qualitativos, as o/a prisma-ótica-olhar-concepção translacional e a pesquisa de enfermagem tem o potencial de contribuir para o avanço da ciência-profissão com alcance para além dos aspectos teóricos e epistemológicos, mas na perspectiva de impacto profundo-expressivo-relevante na construção de evidências científicas com ainda mais rigor e confiabilidade em pesquisas qualitativas e, por tanto, para sua credibilidade e aplicabilidade na prática clínica.

Translation concerns the incorporation of research results into the professional clinical practice, emerging as a "translation" of new knowledge, mechanisms, and techniques from scientific production, providing possibilities for care, management, and administration of health activities.^1^^,^^2^ In the field of nursing, this social commitment began in the mid-2000s,^3^ assumed as a challenge in building a praxis consistent with scientific advances in the area. The concept of validity, in turn, is linked to the very notion of scientific knowledge; however, in the nursing field, it carries different connotations. Its multiple interfaces include its applicability in qualitative research, where the interpretation of validation goes beyond the literal sense of the word, making it closer to the translation of knowledge.^4^ In qualitative studies, validity is considered an adaptable construct capable of facilitating a more reliable approach to the subjectivity of the target audience and enabling the understanding of individual and collective experiences at various levels of depth.^4^^,^^5^

Validity in qualitative research can occur in a preliminary (research formulation), internal (research development), and external (research results) manner.^4^ Therefore, the implicit need for the application of the validity framework is presumed when indicating, through translation, the researcher's implication and their influence on the research scenario as an essential aspect in qualifying the study and safeguarding the dissemination of knowledge as evidence applicable to problem resolution.^6^ Although translational theory can apply to qualitative research, it is crucial to emphasize that other theoretical frameworks also support critical thinking and methodological foundation in the field to underpin the validity of this approach. Accordingly, positivist and interpretative thoughts are delineated. For the former, validity would be an objective attribute, subject to repetition, experimentation, and generalization. Regarding the interpretative conception, the concept facilitates the understanding of the phenomenon, description, and researcher-participant interaction (transactional validity) or the impact of the research on the individual (transformational validity).^7^ Therefore, it approaches the research participant, bringing forth the applicability of knowledge constructed in the context of attention and care practices.

More specifically, qualitative nursing research can be observed from the perspective of historical and conceptual paradigms based on its construction. The perceived view of science, also called the interpretative paradigm, became incorporated into the field during the 1960s, under the aegis of the post-modernism movement, "due to examining phenomena in context, phenomenology, and other perceived views of philosophy leading to the discovery and development of nursing's inherent knowledge".^8^ It is understood how this type of research influenced sociopolitical and educational issues in care, as well as the bases, concepts, and foundations of nursing beyond its practice.^9^

The adoption of the triangulation technique is acknowledged among the processes and procedures employed by researchers. This technique serves as an alternative for exposing researchers to different strategies in order to formulate a viable validation protocol. This can refer to data collection (divergent sources), researchers (consensus), theories (various scientific knowledge), environmental factors (different locations), and methodological approaches (mixed methods).^4^^,^^10^ Within the domain of nursing science, various methods and techniques are integrated, including documentary and bibliographic research, observational surveys, focus groups, individual and in-depth interviews. Additionally, the collection of expert opinions from diverse perspectives related to the study object is incorporated. These methodologies collectively contribute to the construction of specialized care spaces.^11^ These operationalization models are interconnected with the translation of knowledge and align with strategies such as Integrated Knowledge Translation, focusing on collaborative approaches through collective work with knowledge consumers (patients, managers, and healthcare providers). Hence, relational dialogue is encouraged, resorting to knowledge-to-action and maximizing its applicability.^2^

**Figure 1:**
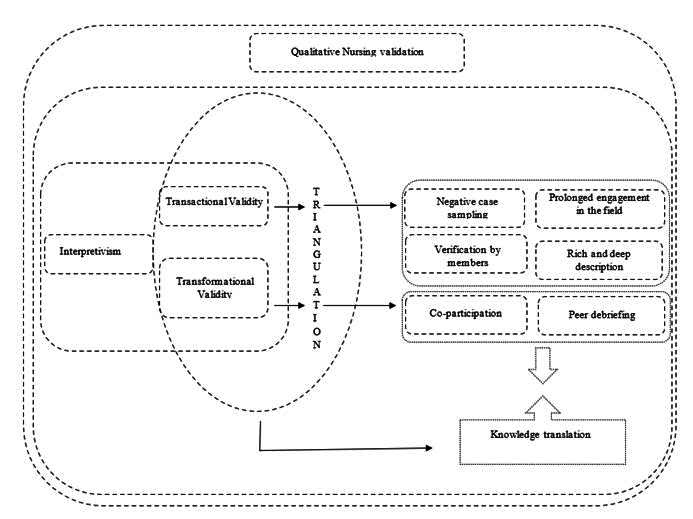
Theoretical-conceptual model for qualitative nursing research validation. Redenção, CE, Brazil, 2022.

Considering the qualitative research approaches and, especially, the validation methods used in nursing, the creation of a conceptual model illustrating consolidated strategies in the field of study, with subsequent contextualization of the method, is deemed important, as depicted in Figure 1.

## Source: developed by the authors.

In the field of nursing, knowledge translation still poses a challenge to the care domain, especially due to the need for the theoretical application of practical constructs in the science of caring. To address this, one must identify the problem and its communication locus among users, develop and select a type of research, analyze its context, and devise ways to apply the solution through activities and interventions.^12^^,^^13^ Some alternatives for such investments include negative case sampling, verification by members, prolonged engagement in the field, case studies, and peer debriefing, which will be addressed in this reflection. The negative case sampling validation strategy is employed in cases where an alternative interpretation is sought, sometimes distinct from what was expected from the research participants. For instance, when women refrain from reporting their aggressors even in situations of violence, a circumstance where reporting would be anticipated.^14^ This type of knowledge provides the basis for constructing safe nursing care.^12^

In the verification by members technique, validation is performed by cross-checking participants' responses and returning the transcript of what was collected/recorded to them.^15^ Through this strategy, knowledge translation in research can be actualized, as what is feasible for the study is validated by its members or external evaluators, thereby integrating practices in health/nursing.^6^ Prolonged involvement in the setting and co-participation of individuals sharpen the sensitivity of the nursing researcher regarding the research locus, reducing discrepancies between the participant's needs and the researcher's thoughts or propositions.^16^ This validation method can be recognized in the realm of health practices to foster surveillance and innovation in knowledge translation.^17^

The case study is also considered a validation strategy due to its rich and deep description of the situation experienced. Through it, a sequence of client identification can be followed, bringing the clinical closer to the practical and proposing facilitated interventions. In its steps, internal, external, and construct validation can be achieved, providing the production of a narrative of the behaviors adopted.^18^ An example of applying the case study method is understanding how nursing care affects the user's ability to regain autonomy, examining the entire care plan and its effectiveness in the individual's rehabilitation or deterioration.^19^ This type of validation is a way to apply knowledge translation as praxis by actualizing the clinical-care experience to the fidelity of the meticulous relationship of its variables.

Finally, the peer debriefing procedure is highlighted as an alternative used in the nursing field in realistic simulations. It involves validation by a third-party evaluator of the content shared by the study participants in the debriefing. This acts as a moderator of what was actually applied, instructing students and enhancing their gains.^20^ This process is part of/is linked to an advanced proposal for knowledge translation associated with a conception for problem resolution in a strategic, enlightening, and deliberative manner.^21^ Therefore, through knowledge of the validation of studies in qualitative research, the need to deepen the theme and research with this approach, emphasizing the application (translation) of knowledge, is emphasized. In this way, the variables relevant to the singularities of each user will be observed in-depth, with the capacity to exceed the strictly scientific and abstract the subjectivity of that which is not merely scientific but rather the lived experience of a social subject.
